# UltraPse: A Universal and Extensible Software Platform for Representing Biological Sequences

**DOI:** 10.3390/ijms18112400

**Published:** 2017-11-14

**Authors:** Pu-Feng Du, Wei Zhao, Yang-Yang Miao, Le-Yi Wei, Likun Wang

**Affiliations:** 1 School of Computer Science and Technology, Tianjin University, Tianjin 300350, China; PufengDu@gmail.com (P.-F.D.); wzhao_cstju@yeah.net (W.Z.); miaoyangyang1998@163.com (Y.-Y.M.); weileyi@tju.edu.cn (L.-Y.W.); 2 School of Chemical Engineering, Tianjin University, Tianjin 300350, China; 3 Institute of Systems Biomedicine, Beijing Key Laboratory of Tumor Systems Biology, Department of Pathology, School of Basic Medical Sciences, Peking University Health Science Center, Beijing 100191, China

**Keywords:** pseudo-amino acid compositions, pseudo-k nucleotide compositions, extensible software

## Abstract

With the avalanche of biological sequences in public databases, one of the most challenging problems in computational biology is to predict their biological functions and cellular attributes. Most of the existing prediction algorithms can only handle fixed-length numerical vectors. Therefore, it is important to be able to represent biological sequences with various lengths using fixed-length numerical vectors. Although several algorithms, as well as software implementations, have been developed to address this problem, these existing programs can only provide a fixed number of representation modes. Every time a new sequence representation mode is developed, a new program will be needed. In this paper, we propose the UltraPse as a universal software platform for this problem. The function of the UltraPse is not only to generate various existing sequence representation modes, but also to simplify all future programming works in developing novel representation modes. The extensibility of UltraPse is particularly enhanced. It allows the users to define their own representation mode, their own physicochemical properties, or even their own types of biological sequences. Moreover, UltraPse is also the fastest software of its kind. The source code package, as well as the executables for both Linux and Windows platforms, can be downloaded from the GitHub repository.

## 1. Introduction

Over the last two decades, huge numbers of biological sequences have been deposited in public databases. Until today, the number of these sequences is still increasing exponentially. However, the cellular and functional attributes of these sequences, no matter whether they are nucleotide sequences or protein sequences, remain largely unknown. It is a very important task for computational biology to predict the functional and cellular attributes of these sequences.

In the view of machine learning, most of these prediction tasks can be formulated as pattern classification problems. As elaborated in a series of publications [1,2,3,4,5,6,7,8], one of the most challenging parts is to represent a biological sequence with a fixed-length numerical vector, yet still keep a considerable amount of the sequence-order information. This is because almost every existing algorithm for these tasks can only handle fixed-length vectors, but not the sequences.

For protein and peptide sequences, Chou proposed pseudo-amino acid compositions (PseAAC) [9] and amphiphilic pseudo-amino acid compositions (AmPseAAC) [10]. Ever since the concepts of pseudo-factors were introduced, they have rapidly penetrated into almost every area of computational proteomics [11,12,13,14,15,16,17,18,19,20]. As elaborated in a review article, the form of classic pseudo-amino acid compositions has been generalized to contain various types of information [21], which is known as the general-form pseudo-amino acid compositions. The applications of PseAAC concepts have been summarized in the review papers [22,23].

Recently, the concept of PseAAC has been extended to represent nucleotide sequences [24]. Chen et al. developed pseudo-dinucleotide compositions (PseDNC) to predict DNA recombination hostspots [25]. This formulation was then extended as pseudo-k nucleotide compositions (PseKNC), which have been applied in predicting splicing sites [26], predicting translation initiation sites [27], predicting nucleosome positions [28], predicting promoters [29], predicting DNA methylation sites [30], predicting microRNA precursors [31] and many others [32,33,34,35,36,37,38,39,40,41].

In the early days of pseudo-amino acid compositions, every study had to implement PseAAC independently. Although the algorithms in every implementation are identical, different implementations may introduce computational discrepancies due to technical details. For example, different implementations may give results with different precisions. This kind of differences may be amplified by machine-learning based predictors, which may eventually produce different prediction results. For another example, different implementations may have very different computational efficiencies. This means one implementation may only use a second to process a dataset, while another program may require over an hour to achieve the same results on the same dataset with the same parameters.

To solve these problems, a universal implementation of the algorithm should be provided. Many efforts have been made for this purpose [42,43,44,45,46,47,48,49,50,51,52]. The first program focus on the PseAAC formulation is the PseAAC server [43], which was brought online in the year 2008. The PseAAC server can compute Type-I and Type-II PseAAC using six different kinds of physicochemical properties of amino acids. The PseAAC server has a friendly user interface, which is convenient and efficient for small datasets. However, for large datasets and the repeatedly parameter scanning process, the computational efficiency of the PseAAC server is not ideal. The PseAAC-Builder [45], which was released in the year 2012, is dedicated to improving the efficiency. Unlike the PseAAC server, the PseAAC-Builder is a stand-alone program that can be executed locally. It has a simple graphical user interface (GUI) for the users’ convenience. It can also be executed in a command line environment. The computational efficiency of PseAAC-Builder is much higher than the PseAAC server, especially in the command line environment. Although the PseAAC-Builder includes over 500 different types of physicochemical properties, it did not provide the ability to compute general form PseAAC. PseAAC-General [46], which is a major upgrade to the PseAAC-Builder, was developed to solve this problem. PseAAC-General provides the ability to compute several commonly used general forms of PseAAC, such as the GO mode, the functional domain mode and the evolutionary mode. The users of PseAAC-General can slightly extend its ability by using Lua scripts.

After Chen et al. proposed the PseKNC representations for nucleotide sequences, similar software and services were needed for DNA and RNA sequences. Chen et al. released the PseKNC [48] and PseKNC-General [49] packages for converting DNA/RNA sequences into its PseKNC or general form of PseKNC representations. Liu et al. developed the repDNA [50], repRNA [51], and Pse-In-One [52] services for more types of descriptors. The Pse-In-One service attempts to be a universal online service that can be applied on both protein and nucleotide sequences.

However, all existing software packages and online services suffer from three problems. (1) Lack of extensibility. Most of the existing software can only be used to produce existing modes of representation. The users cannot extend the software to handle their own novel representation modes. Although PseAAC-General can be extended by using Lua script, it can only be used for protein sequences; (2) Lack of flexibility. Most of the existing software can only handle one type of biological sequences, either nucleotide sequences or protein sequences. Pse-In-One is the only existing service that can handle protein sequence as well as nucleotide sequences. However, no program can handle user-defined sequence types. For example, when studying the protein phosphorylation sites, the modified residues should have different notations of sequences, which are not in the standard 20 letters. The users need to define the extra letters to represent the modified residues. As far as we know, no program can handle this kind of sequence; (3) Lack of computational efficiency on large datasets. Most of the existing programs are not designed to handle large datasets. They may need many minutes to process a million sequences. If a user needs to repeatedly scan parameters of a representation, the processing time may be days or even weeks.

In this paper, we proposed the UltraPse program, which is a universal and extensible software platform for all possible sequence representation modes. The UltraPse program unified the processing of nucleotide and protein sequence in one program, as well as the user-defined sequence types. UltraPse supports two forms of extension modules, the BSOs (Binary Shared Objects) and the Lua scripts, which are called the TDFs (Task Definition Files) in UltraPse. The users can develop their own modes by just writing several lines of Lua scripts. UltraPse has very high computational efficiency. It is even faster than the PseAAC-General, which used to be the fastest program of its kind. For the users’ convenience, we have integrated many existing modes within the UltraPse. We expect that the UltraPse program can be a useful platform which simplifies all future programming works in developing novel sequence representation modes. All source codes of UltraPse, including some extension modules can be downloaded freely under the term of GNU GPL (GNU General Public License) v3 from the GitHub repository: https://github.com/pufengdu/UltraPse.

## 2. Results and Discussion

### 2.1. Computational Efficiency Analysis

**Figure 1:**
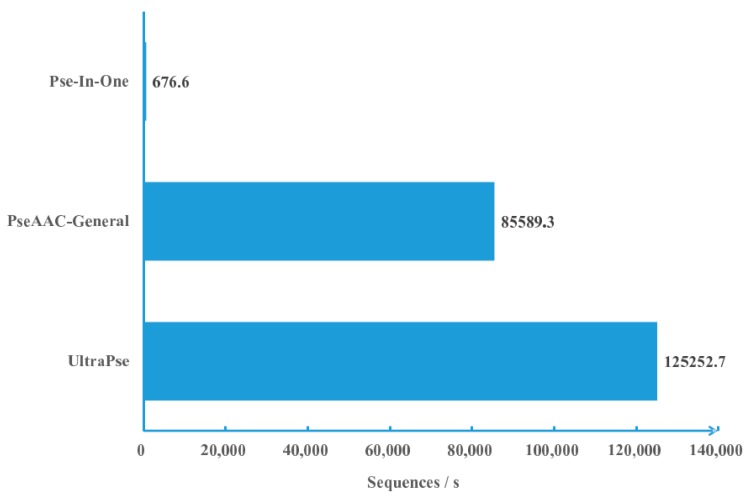
Computational efficiency comparisons. Three programs are compared. The comparison was carried out by letting the three programs compute amino acid compositions on the same dataset on the same machine. Every program was executed with the same parameters for three times. The average execution time was applied in calculating the computational efficiency. The computational efficiency is measured by the average number of sequences that are processed every second. Pse-In-One: A program in literature [52]; PseAAC-General: A program in literature [46]; UltraPse: A program of this work.

We compared the computational efficiency of UltraPse to that of PseAAC-General and Pse-In-One under the same conditions. As in Figure 1, the UltraPse can process over 120 thousand sequences per second, while PseAAC-General can process about 85 thousand sequences per second. Unfortunately, the Pse-In-One can process only less than one thousand sequences per second. According to these results, the computational efficiency of UltraPse is roughly 1.5 times of the PseAAC-General, and about 185 times of Pse-In-One. Since the algorithms of the three programs are essentially the same, the reason for the efficiency differences resides in the technical details of the implementations.

### 2.2. Flexibility and Extensibility

**Figure 2:**
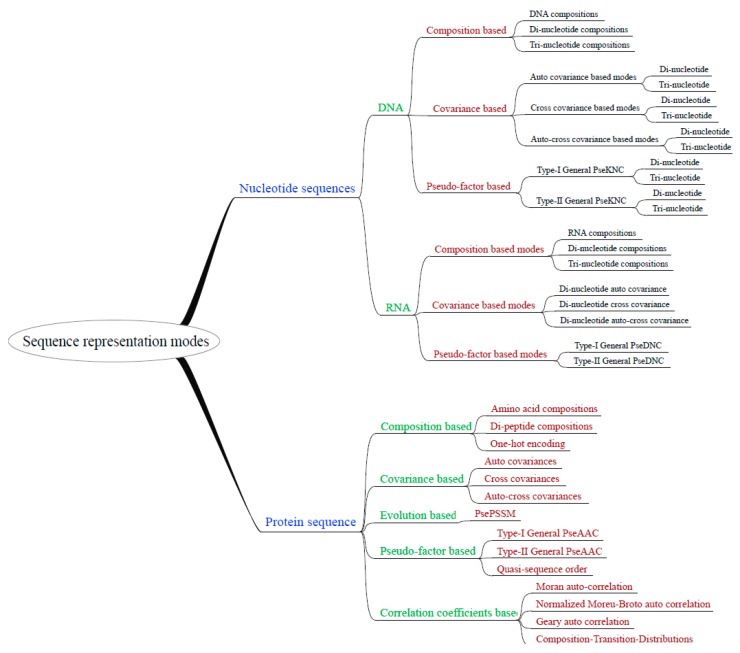
Hierarchical organization of integrated sequence representation modes. UltraPse integrated the sequence representation modes in its distribution package. Most of these modes can also be applied in user-defined sequence types, as long as the users provide proper definitions of the physicochemical properties.

We integrated 35 sequence representation modes within the UltraPse. The representation modes can be organized hierarchically as in Figure 2. The integrated modes can be used to represent protein, as well as DNA and RNA sequences. The modes cover most of the representation modes that can be generated by PseAAC-General, PseKNC-General, and Pse-In-One. Moreover, UltraPse can generate even more modes, for example, the commonly used one-hot encoding mode [53,54,55]. The sequence representation modes of UltraPse can be extended by using BSOs and TDFs. According to our own works, using UltraPse in developing novel representation modes can save over half of the programing labor.

Besides the user-defined representation modes of protein and nucleotide sequences, the users of UltraPse can define their own sequence types using TDFs. They are allowed to choose a set of letters other than the standard ones to represent additional information. For example, a user can use C for cytidines on a DNA sequence, and M for methylated cytidines. The choice of the letter M totally depends on the users. Even more, the users of UltraPse can define their own physicochemical properties with TDFs.

The TDFs of UltraPse is written using Lua language, which is a simple, powerful and extensible programming language which has been applied in bioinformatics software previously [56]. We provide over 20 UltraPse specific functions and interfaces. Users can access and modify UltraPse internal data structures using these functions in TDFs. We compared the flexibility and the extensibility of different software in Table 1.

### 2.3. Compatibility and Robustness

UltraPse can recognize FASTA format files that are directly downloaded from one of the following five databases: GenBank, UniProt, EMBL, DDBJ, and RefSeq. The sequence identifiers and comments in these public databases can be automatically recognized. For FASTA file that are not from these public databases, UltraPse can also recognize them as long as the comment line of every sequence is unique in the FASTA file. Besides the FASTA format requirements, there is no additional restriction on input data format. As indicated in Table 2, this is a unique advantage of UltraPse.

According to Chou’s five step rule [12,21,57,58,59,60], before converting biological sequences into numerical vectors, a high-quality benchmark dataset must be constructed. The construction of a dataset usually includes a step to filter out the sequences containing non-standard letters. For example, B, J, or X appear in protein sequences in the UniProt database. However, the sequences containing these letters are hardly suitable for further analysis in many cases. As indicated in Table 2, UltraPse provides a user-controllable data fault tolerant ability. According to users’ choice, when one of these sequences is encountered, UltraPse can automatically skip the sequence or abort all further computations. This function is useful in adopting third-party datasets in practical works, because filtering out the sequences usually requires tedious programming work.

### 2.4. Technical Detail Comparison

Most state-of-the art software is written in Python, while PseAAC-General and UltraPse are written in C++. This difference eventually made the difference in computational efficiency. Since the computational efficiencies of PseAAC-General and UltraPse are comparable, we can compare several technical details of them.

PseAAC-General is a program that can be extended by using Binary Extension Modules (BEMs). However, it should be noted that, the BEMs of PseAAC-General are completely different to the BSOs in UltraPse. A BEM of PseAAC-General is just a compressed data block. However, how this data block should be used, was still implemented by the PseAAC-General main program. In the UltraPse, a BSO is actually a dynamically loaded library, which contains all the information and instructions for constructing one or more sequence representation modes. Therefore, the BSOs of UltraPse are much more flexible than the BEMs of PseAAC-General.

We have seen that UltraPse has roughly 1.5 times the efficiency of PseAAC-General. This advantage is achieved by an internal representation scheme and a pre-computing mechanism of UltraPse. In PseAAC-General, the sequences are converted to a series of physicochemical properties. The sequence descriptors are then computed according to the corresponding algorithms. However, this intuitive implementation requires repeatedly computing dot-product or Euclidean distance between physicochemical vectors of different amino acids. Since the combination of two different amino acids is limited, we pre-compute the dot-product and Euclidean distance for all possible combinations in UltraPse. The sequences in UltraPse are not converted into a series of physicochemical properties. They are converted into UltraPse internal indices, which can be used to quickly find correct values that have been pre-computed. When computing only the amino acids compositions, the implementations of PseAAC-General and UltraPse are similar. However, UltraPse still benefits from converting all sequences into internal indices first. Because, the amino acids counting procedure becomes simpler, this allows the compiler to do more optimization for speed. This is why UltraPse is faster than PseAAC-General.

### 2.5. Future Works in Plan

Besides the practical application of UltraPse program in research projects, there is still much work to do in terms of software development. The work at first priority is to add an automated unit-testing facility in the source code of UltraPse. Unit-testing is good practice in software engineering to ensure robustness of large scale software. It will be very important for the future versions of UltraPse. The next work in plan is to enable UltraPse support more data formats as input files. As far as we can tell, no existing program in representing biological sequences can handle file formats other than FASTA. We will make the next version of UltraPse handle FASTA, FASTQ, and several other formats of input file.

### 2.6. Availability

The UltraPse software is provided as source codes and binary packages. All the source codes can be downloaded from the GitHub repository (Available online: https://github.com/pufengdu/UltraPse). The binary distribution packages can also be downloaded from the Release sub-directory in the GitHub repository. Currently, there are binary packages for Windows and Linux platforms. The Windows binary program can be executed directly. The Linux binary package has been tested on a freshly installed Ubuntu Linux Server 16.04.3.

## 3 Methods

### 3.1. Efficiency Comparison Protocols

We performed computational efficiency comparisons on a server with an Intel Xeon X3470 processor and 32 GB memory. To perform a fair comparison, we installed Pse-In-One locally on the server. We also locally compiled and installed PseAAC-General and UltraPse on the same server. The testing dataset is the “huge” testing dataset that can be obtained from the official website of PseAAC-General. This dataset contains 516,081 protein sequences. Since the Pse-In-One keeps complaining about non-standard letters and too short sequences in the dataset, we excluded all the sequences that have non-standard letters. The remaining 513,536 protein sequences were fed into three programs independently. All three programs are configured to compute only amino acid compositions. The computational times are measured by the “real” time value of the standard Linux time command. To eliminate random errors, every program was executed consecutively with exactly the same configuration three times. The average computational time was used in calculating computational efficiency.

### 3.2. Abstracted Software Design

**Figure 3:**
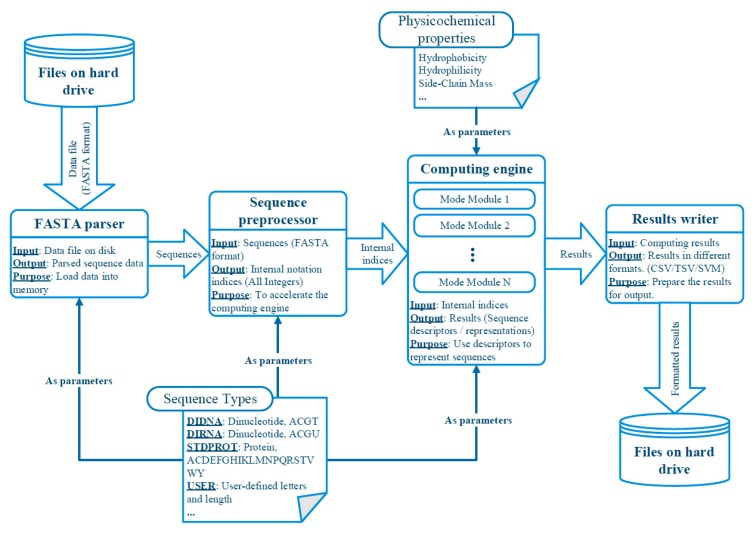
The abstracted software design and data flow chart of UltraPse.

We illustrate the internal structure and the data-flows of UltraPse in Figure 3. There are four major parts within UltraPse. They are the FASTA parser, sequence preprocessor, computing engine, and the result writer. The FASTA parser is responsible for loading FASTA format sequences into the memory from a hard drive. It also organizes the sequences according to their identifiers and their sequence types. These sequences are then sent to the sequence preprocessor, where the sequences are converted to UltraPse internal indices according to the sequence type definitions. The computing engine is composed of several mode modules, which are configured according to user requirements. The internal indices go through all mode modules. Eventually, sequence descriptors are generated. The result writer exports these descriptors on the hard drive according to the format requirements.

### 3.3. Implementation Technology

The UltraPse main program is written using standard C++ language, following C++14 standard. The destination hardware architecture is x86-64. The dependencies of the UltraPse main program include GNU standard C library and the embedded interpreter of Lua scripting language. The BSOs of UltraPse are also written using C++, following the same rules as the main program.

On the Linux platform, the compiler for producing binary executables is the GNU g++ version 7.2. The users should first install Lua scripting language. The configuration and compilation of UltraPse need the library provided by the Lua package. On the Windows platform, the MinGW64 version g++ compiler is applied. Several independent libraries are required to compile the codes. For the convenience of Windows users, we provide a binary executable package for the Windows platform.

The TDFs are provided as platform-independent Lua scripts, which can be viewed, edited, and loaded as their original form. The internal data structures of UltraPse can be accessed by Lua scripts using UltraPse specific functions and interfaces. The details on how to write TDFs can be found in the software manual.

### 3.4. A Practical Example

**Figure 4:**
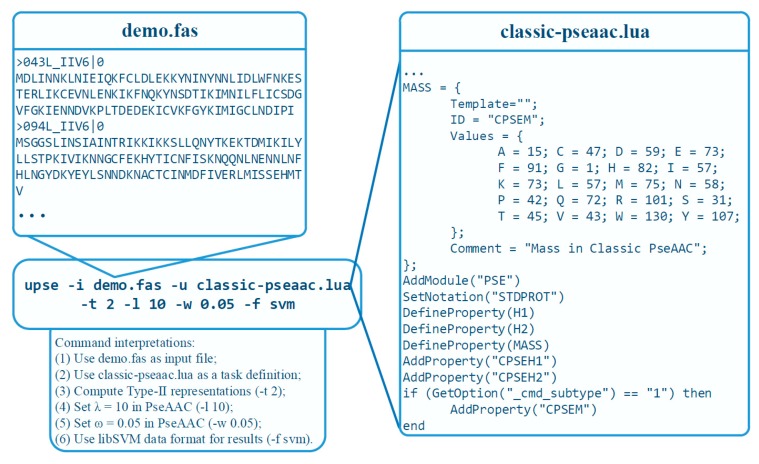
An example on using UltraPse. UltraPse was used to implement classic pseudo-amino acid compositions. A TDF: classic-pseaac.lua, was applied. The FASTA format sequences are stored in the demo.fas file. The command options indicate that the Type 2 PseAAC will be computed with parameters: λ = 10 and ω = 0.05. The output format is compatible to libSVM.

Figure 4 demonstrate a practical example. The classic pseudo-amino acid composition modes, including type-I and type-II, are implemented using a TDF in UltraPse. The TDF for classic pseudo-amino acid compositions can be found in the “tdfs” subdirectory of UltraPse. The right part of Figure 4 is a part of this TDF. With this TDF, the users only need to specify some parameters on the command line. For example, the “–l 10 –w 0.05” on the command line indicate the value of λ and ω in the PseAAC formulations. Unlike PseAAC-General, where the meanings of all command line options are fixed, the meanings of command line options can be altered by the TDFs in the UltraPse. This is to simplify the development of novel sequence representation modes, where parameters are required to perform correct and efficient computations.

## 4. Conclusions

In this paper, we described our new software, the UltraPse (Available online: https://github.com/pufengdu/UltraPse). UltraPse is a universal and extensible software platform for generating biological sequence representations. Since many programs have already been released for various sequence representations, UltraPse has no intention to be a new competitor on the same playground. We expect that UltraPse can work side-by-side with other existing programs, such as PseAAC-General, PseAAC-Builder and Pse-In-One, to accelerate the process of generating sequence representations under various working environments. 

Although we have integrated many existing sequence modes within the UltraPse, it should be noted that the major advantage of UltraPse is its flexibility and extensibility. It was designed to be a software platform rather than a program with specific functions. It aims at simplifying all future programming works in developing novel sequence representations. 

Web servers have already been proved to be a good method in releasing software. However, presenting UltraPse with a web server will severely damage its computational efficiency. Therefore, we do not provide an online web server for UltraPse. We would rather provide it as a local program. The users need to compile and install it on their own servers. The graphical user interfaces (GUI) is useful on platforms like Microsoft Windows. We will develop a GUI for UltraPse on the Windows platform in future.

## Figures and Tables

**Table 1:** Software function comparison in terms of flexibility and extensibility.

Software Functions	Sequence Types	Extensibility
UltraPse	DNA, RNA, Protein, User-defined types	Users can define their own sequence types, representation modes and physicochemical properties
PseAAC-General [46]	Protein	Users can define their own representation modes
PseAAC-Builder [45]	Protein	No extensibility
Pse-In-One [52]	DNA, RNA, Protein	Users can define their own physicochemical properties
PseKNC [48]	DNA, RNA	Users can define their own physicochemical properties
PseKNC-General [49]	DNA, RNA	Users can define their own physicochemical properties

**Table 2:** Software function comparison in terms of data processing ability.

Software	Output Formats	Input Formats	Data Fault Tolerant ^a^
UltraPse	SVM ^b^, TSV ^c^, CSV ^d^	Multi-line FASTA (Automatic ID recognition for UniProt, GenBank, EMBL, DDBJ and RefSeq)	User-controllable behavior on data faults
PseAAC-General [46]	SVM, TSV, CSV	Single-line FASTA (With restrictions on comment line) ^e^	Automatically ignore and report data faults
PseAAC-Builder [45]	SVM, TSV, CSV	Single-line FASTA (With restrictions on comment line)	Automatically ignore and report data faults
Pse-In-One [52]	SVM, TSV, CSV	Mutlti-line FASTA	Abort processing on data faults
PseKNC [48]	SVM, TSV, CSV	Mutlti-line FASTA	Abort processing on data faults
PseKNC-General [49]	SVM, TSV, CSV	Mutlti-line FASTA	Abort processing on data faults

^a^ Data fault tolerant: The behavior of a software when it encounters some invalid data records. Here, the invalid data records include the sequences with non-standard letter and the sequence without sufficient length; ^b^ SVM: data format for libSVM [61]; ^c^ TSV: tab separated vector; ^d^ CSV: comma separated vector; ^e^ Single-line FASTA: the sequence of a record in the file must not spread to multiple lines. Both PseAAC-General and PseAAC-Builder have the same restrictions.

## Abbreviations

AmPseAAC	Amphiphilic pseudo amino acid composition
BEM	Binary extension module
BSO	Binary shared objects
GPL	General public license
GUI	Graphical user interfaces
PseAAC	Pseudo-amino acid composition
PseDNC	Pseudo-dinucleotide composition
PseKNC	Pseudo-k nucleotide composition
TDF	Task definition file
